# Optimization of the nutritional constituents for ergosterol peroxide production by *Paecilomycescicadae* based on the uniform design and mathematical model

**DOI:** 10.1038/s41598-022-09773-x

**Published:** 2022-04-07

**Authors:** Shi Fenhui, Linfu He, Jingya Qian, Zhicai Zhang, Huihua Zheng

**Affiliations:** 1 School of Food Science and Biological Engineering, Jiangsu University, Zhenjiang, 212013 People’s Republic of China; 2 Institute of Agro-Production Processing Engineering, Jiangsu University, Zhenjiang, 212013 People’s Republic of China; 3 Zhenjiang Yemaikang Food Bio-Technology Co., Ltd, Zhenjiang, 212013 People’s Republic of China; 4 Key Laboratory of Edible Mushroom Processing Ministry of Agriculture and Rural Affairs, Jiangsu Alphay Biotechnology Co., Ltd, Nantong, 226009 People’s Republic of China

**Keywords:** Biotechnology, Fungi

## Abstract

we optimized medium components for the production of ergosterol peroxide (EP) by Paecilomyces cicadae based on a mono-factor experiment, a uniform design, and a non-linear regression analysis. The maximum EP yield achieved was 256 μg/L, which was increased by 5 folds compared with that before the optimization. Structured Monod model, Andrews model, Contois model, and Aibe model were developed to describe the effects of viscosity inhibition, substrate, and production on biomass growth. The results showed that the Monod model could predict biomass growth, and the effects of viscosity and substrate on the EP concentration were significantly higher compared with the effect of production. The addition of water and glycerol could decrease the viscosity inhibition and glycerol inhibition, and further increase the EP yield. The newly developed structured model was demonstrated for batch growth of *P.* *cicadae*.

## Introduction

Ergosterol peroxide (EP: 5ɑ, 8ɑ-epidioxy-22E-ergosta-6, 22-dien-3β-ol) possesses a wide spectrum of biological properties, such as anti-oxidant^1^, anti-inflammatory^2,3^, immune-modulating^4,5^, and anti-tumor activities, and it can inhibit the activation of renal fibroblasts induced by TGF-1^6–8^. EP mainly comes from the fruiting bodies of medicinal fungi, including *Paecilomyces* *cicadae*^8^, *Inonotus* *obliquus*^9^, *Sarcodon* *aspratus*^7^, *Ganoderma* *lucidum*, and *Hericium* *erinaceus*^10^, as well as some yeasts^10^. *Paecilomyces* *cicadae* is a fungal strain belonging to the *Paecilomyces* genus of ascomycete fungi. In the *Paecilomyces*, 400 species have been described^11^. *P.* *cicadae* has been used in Chinese herbal medicinal prescriptions to treat many types of diseases, including kidney disease, for thousands of years^11–13^. *P.* *cicadae* has recently received increasing attention due to its reno-protective activity^14–16^. In our previous study, EP can be isolated and purified from the mycelia of *P.* *cicadae*^17^*.* EP derived from mycelia can be used to treat renal cell carcinoma in vitro through multiple mechanisms, including suppressing cell growth, colonization, migration, and invasion, inducing cell cycle arrest, attenuating β-catenin pathways, and triggering apoptosis^17^.

Although many types of food and medical fungi can synthesize EP, large-scale production of EP has not been reported. The bottleneck restricting the mass production of EP can be attributed to the extremely low yield of EP. The strategies to increase the yield of fermentation products include screening and breeding of strains and optimization of culture conditions and medical components. The yields of metabolite and biomass are strongly affected by medium composition and cultivation conditions. The medium composition includes carbon sources, nitrogen sources, and trace elements. The cultivation conditions include aeration volume, agitation speed, pH, incubation time, and temperature. Therefore, to increase microbial production with less economic cost, these factors need to be simultaneously optimized. The surface response method, orthogonal design, and uniform design are effective methods to optimize multivariates^18–23^. As a widely used experimental design of measurement uniformity, the uniform design method is first proposed by mathematicians of Wang and Fang in 1978. The basic idea of the uniform design is to arrange the test points at a uniform distribution within the scope. The uniform design is suitable for the test with multiple factors and multiple levels, and it can effectively reduce the cost of the experiment to improve the research efficiency^24,25^.

Modeling of fermentation kinetics is widely used to control, predict, optimize, and design fermentation processing. All kinetic models of fermentation can be divided into unstructured and structured models. Both types of models are studied to explain the kinetics of biomass and fermentation reactions^26–29^. The unstructured model (logistic model) applied to several fermentation processes of filamentous fungi may be very useful as data fitters^30,31^. A great limitation of the unstructured model is that these models are mainly composed of empirical equations, which cannot reveal the mechanisms hidden behind the observed phenomena. The modeling of fungal growth through its biomass is especially interesting for the control of fermentation processes. Therefore, it is necessary to develop more suitable models to provide a better understanding of the mechanisms, which regulate the fermentation process. Although many structured models in the literature fit the microbial growth data well, such as the Monod model, Andrews model (inhibition model by substrate), Contois model (inhibition model by viscosity), and Aibe model (inhibition model by production), these models have not been reported to fit the fermentation process of filamentous fungi.

In the present study, we aimed to optimize the nutritional constituents for *P.* *cicadae* to increase the EP yield. The optimization strategy included: (1) the primary nutritional requirement for EP synthesis was achieved by classical mono-factor design; (2) the interaction between nutritional components was achieved by uniform design and statistical analysis; and (3) the interaction between nutritional components was evaluated based on four structured kinetic models.

## Results and discussion

### Mono-factor experimentation

#### Effect of carbon source

**Figure 1:**
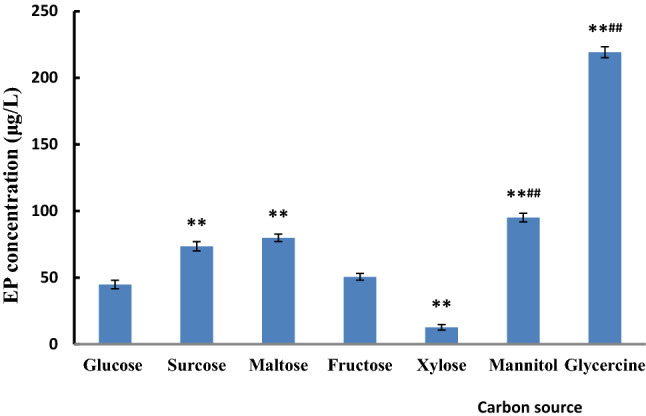
Effect of carbon sources on the EP concentration. **p ≤ 0.01, compared with the glucose using carbon sources; ^##^*p* ≤ 0.01, compared with the maltose, sucrose, and fructose using as carbon sources, respectively. The other components of THE medium were the same, and the culture was carried in a rotary shaker at 25 °C and 120 rpm for 48 h.

The carbon source is a major component of the cytoskeleton in the process of fungal growth, and it provides the energy for biomass growth and metabolite synthesis. Saccharide is the main carbon source for the synthesis of biomass and product in the fungal growth process. Most fungi can grow and synthesize the metabolite in the medium using sucrose, glucose, or maltose as carbon sources^32^. The effect of carbon sources on EP synthesis is rarely reported in the literature. In the present study, glucose, sucrose, maltose, fructose, mannitol, and glycerol were used as carbon sources to study the effects of various types of carbon sources on the EP synthesis in the fermentation process of *P.* *cicadae*. Figure 1 shows that the EP yield was significantly higher when glycerol or mannitol was added into the fermentation broth compared with the other carbon sources (219 and 95.09 μg/L), respectively. The EP yield was significantly higher when the disaccharides (maltose and sucrose) were added compared with monosaccharides (glucose and fructose), while the EP yield between maltose and sucrose or between glucose and fructose was not significantly different.

Carbon source is the main source of nutrients and energy in the process of mycelial growth and metabolite synthesis. Therefore, the type and concentration of carbon source directly determine the specific growth rate, the consumption rate of carbon source, and the synthesis rate of metabolites. In the process of filamentous fungal fermentation, many studies have investigated the relationship between the yield of secondary metabolite and carbon sources. Generally, most organisms prefer to utilize glucose as a carbon source. However, glucose is not conducive to the synthesis of metabolites. The phenomenon can be attributed to the repression of carbon catabolites. Glucose, for example, can inhibit antibiotic synthesis by blocking or inhibiting key enzymes in the antibiotic biosynthetic pathway^33,34^. As a carbon source, glycerol or mannitol can avoid the inhibition of carbon catabolism. The effect of membrane fatty acids on permeability has been reported^35^. Few works have investigated the influence of the membrane fatty acids of *R.* *arrhizus* cell on permeability. Different components of the culture medium can change membrane composition and lead to the change of cell membrane permeability. The ratio of saturated and unsaturated fatty acids directly determines the permeability of the membrane. Glycerol has been proven to increase saturated fatty acids of cell membrane^36^. Fatty acid saturation determines the overall stability of the membrane to some extent, while an increase in fatty acid saturation can cause a decrease in membrane fluidity and thus its permeability^37^, and it blocks the secretion of ergosterol, the precursor of EP, and promotes the synthesis of EP. Therefore, glycerol was chosen as the carbon source in the subsequent experiments.

#### Effect of nitrogen source

**Figure 2:**
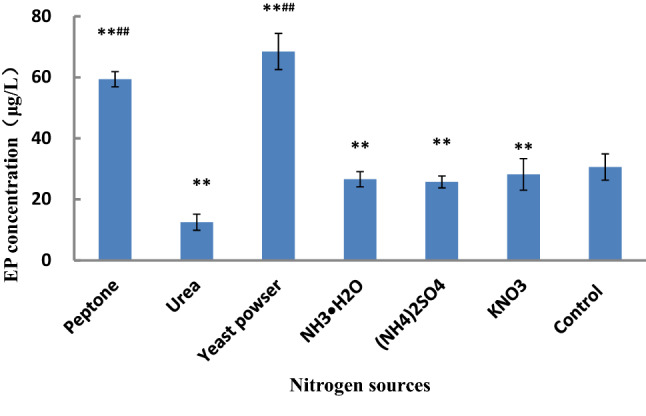
Effect of nitrogen sources on the EP concentration. The control was the medium without nitrogen sources. **p ≤ 0.01, compared with the control group; ^##^*p* ≤ 0.01, compared with the groups using NH_3_∙H_2_O, (NH_4_)_2_SO4, and KNO_3_ as nitrogen sources, respectively. The other components of the medium were the same, and the culture was carried in a rotary shaker at 25 °C and 120 rpm for 48 h.

Many fungi can utilize a wide variety of nitrogen sources. The effects of different nitrogen sources on mycelial growth and product synthesis are very different^38^. Many nitrogen sources are involved in the synthesis of nitrogen-containing metabolites, such as amino-acid, nucleotides, and vitamins. These amino acids and nucleotides are the precursors of some secondary metabolites^39^. Therefore, nitrogen sources must meet the needs of mycelial growth and the synthesis of some metabolites. Nitrogen sources can be divided into organic and inorganic nitrogen sources. Peptone, soybean meal, corn syrup, beef extract, and yeast powder are the major organic nitrogen sources, and KNO_3_, NaNO_3_, CO(NH_2_)_2_, (NH_4_)_2_SO_4_, and NH_4_OH are the primary inorganic nitrogen sources. Peptone, yeast powder, KNO_3_, CO(NH_2_)_2_, (NH_4_)_2_SO_4,_ and NH_4_OH were used to study the effect of nitrogen source on the EP synthesis by *P.* *cicadae*, and the experimental results are shown in Fig. 2.

Figure 2 shows that a significant enhancement was observed when utilizing peptone and yeast powder for EP yield. The maximum EP yield (68.49 μg/L) was obtained when the yeast powder was used as the nitrogen source, followed by peptone (59.38 μg/L). Their EP yields were significantly higher compared with the control group without the nitrogen sources (30.6 μg/L). However, after KNO_3_, CO(NH_2_)_2_, (NH_4_)_2_SO_4,_ and NH_4_OH were added, respectively, the mycelia grew slowly. Moreover, after the fermentation, the dried mycelium was dark, and its EP concentration was less than 26.60 μg/L. The results showed that inorganic nitrogen in the fermentation of *P.* *cicadae* was not good for EP production.

The pathway of nitrogen metabolism is closely related to the pathway of secondary metabolite biosynthesis. Nitrogen regulation in fungi has been extensively reviewed^40,41^. One of the differences between yeast powder and peptone is that yeast powder contains higher levels of biotin. The biotin can increase the cell membrane density, reduce the cell membrane permeability, block the secretion of EP precursor ergosterol, and promote the synthesis of EP. This also led to the highest yield of EP when yeast powder was added. However, excessive biotin prevents the transport of the substrate and metabolites between the extracellular and intracellular. To control the content of biotin in the medium, the yeast powder and peptone were chosen as the nitrogen source in the uniform design experiment.

#### Effect of inorganic salt

**Figure 3:**
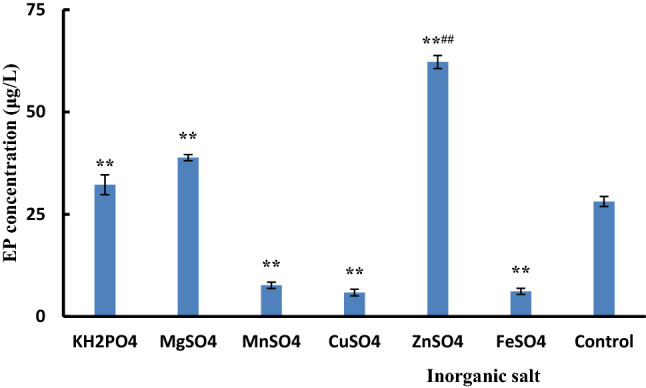
Effect of inorganic salt sources on the EP concentration. The control was the medium without inorganic salt.**p ≤ 0.01, compared with the control group; ^##^*p* ≤ 0.01, compared with the groups when KH_2_PO_4_ and MgSO_4_ were added, respectively. The other components of the medium were the same, and the culture was carried in a rotary shaker at 25 °C and 120 rpm for 48 h.

As the coenzymes of many types of enzymes, inorganic salts play a vital role in the growth and metabolism of microorganisms, the balance of osmotic pressure, and the translocation of nutrients and metabolites between the intracellular and extracellular cells. Therefore, we investigated the effects of six inorganic salts on the EP yield in the broth of *P.* *cicadae*, including KH_2_PO_4_, MgSO_4_, MnSO_4_, CuSO_4_, ZnSO_4_, and FeSO_4_. Figure 3 shows that the EP yield was significantly higher when KH_2_PO_4_, MgSO_4_, and ZnSO_4_ (32.21 ± 2.41, 38.85 ± 0.75, and 62.23 ± 1.60 μg/L, respectively) were added compared with the control group (no addition of inorganic salt, 28.12 ± 2.03 μg/L). Especially, the increase of EP yield was the most significant when ZnSO_4_ was added. ZnSO_4_ is beneficial to the fermentation of *Cordyceps* *jiangxiensis* to produce intracellular polysaccharides and mycelia^42^, which is consistent with our results. The EP yield was significantly low when MnSO_4_, CuSO_4,_ and FeSO_4_ were added. Therefore, we chose KH_2_PO_4_, MgSO_4,_ and ZnSO_4_ as the inorganic salts.

### Uniform-design experimentation

According to the result of the mono-factor experimentation, glycerol, yeast powder, peptone, KH_2_PO_4_, MgSO_4,_ and ZnSO_4_ were screened as the independent variables, and U_10_(10^6^) was applied to investigate the effects of six factors on the EP yield. Table 1 shows the scheme of U_10_(10^6^). The sampling time was selected as the control variable, which was set at 28, 56, 84, and 112 h. Table 1 shows the experimental results.

**Table 1:** The scheme and running results of U_10_ (10^6^) uniform-design (μg/L).

Run	Level (g/100 mL medium)						Results			
	Glycerinol	Yeast powder	Peptone	ZnSO_4_	MgSO_4_	KH_2_PO_4_	28 h	56 h	84 h	112 h
1	4.5	0.5	0.2	0.035	0.06	0.18	28.43	244.67	177.83	44.42
2	3.5	0.2	0.35	0.025	0.24	0.2	40.49	227.96	147.46	53.16
3	5	0.25	0.55	0.005	0.08	0.14	32.85	139.16	139.41	84.86
4	2	0.4	0.25	0.01	0.18	0.12	24.3	195.99	160.97	69.85
5	6	0.3	0.3	0.045	0.14	0.08	28.37	106.63	63.58	48.71
6	2.5	0.35	0.6	0.04	0.12	0.22	33.39	157.66	99.04	64.24
7	5.5	0.45	0.65	0.03	0.22	0.1	34.42	136.68	147.17	99.00
8	3	0.6	0.45	0.02	0.1	0.06	17.21	49.36	105.80	90.09
9	6.5	0.55	0.4	0.015	0.16	0.24	25.3	156.57	108.10	75.87
10	4	0.65	0.5	0.05	0.2	0.16	29.94	192.52	118.74	96.68

To more accurately analyze the relationship between each factor and EP yield, the following formula was used to fit the data in Table 1.

1 $${Y}_{EP}=\sum_{1}^{2}({\alpha }_{i}{F}_{i}+{\beta }_{i}{F}_{i}^{2})+\sum_{3}^{7}{{\alpha }_{i}F}_{i}+C$$

where *F*_*i*_ is the sampling time (T), glycerol concentration, yeast extract concentration, peptone concentration, ZnSO_4_ concentration, MgSO_4_ concentration, or KH_2_PO_4_ concentration. *α*_i_ and *β*_i_ represent the coefficients of factor *F*_*i*_ and *F*_*i*_^*2*^, respectively. *C* represents the fitting constant. Table 2 presents the fitting results. F-value (7.754) and *p*-value (0.000) of the model with 95% confidence in Table 2 implied that the fitted equation was extremely significant and reliable.

**Table 2:** The coefficient of each item and ANOVA results of regression equations with different factors and EP yields.

Item	Co-efficient	t-value	Sig
Constant	− 270.021	− 3.474	0.002
*F*_*1*_^*2*^	− 0.059	− 7.570	0.000
*F*_*1*_	8.617	7.760	0.000
*F*_*2*_^*2*^	− 6.234	− 1.681	0.103
*F*_*2*_	50.605	1.583	0.124
*F*_*3*_	15.765	0.354	0.726
*F*_*4*_	− 43.796	− 0.998	0.331
*F*_*5*_	− 466.113	− 0.983	0.333
*F*_*6*_	135.518	1.204	0.238
*F*_*7*_	177.269	1.641	0.111

Model: F = 7.454, *p* = 0.000.

The coefficients of each item in Table 2 were introduced into Eq. (1) to give the following Eq. (2).

2 $${Y}_{EP}=-270.21-0.059{F}_{1}^{2}+8.617{F}_{1}-6.234{F}_{2}^{2}+50.605{F}_{2}+15.755{F}_{3}-43.796{F}_{4}-466.113{F}_{5}+135.518{F}_{6}+172.269{F}_{7}$$

The coefficients of F_3_, F_6,_ and F_7_ greater than 0 indicated that positive correlations existed between *Y*_*EP*_ and *F*_*3*_, between *Y*_*EP*_ and *F*_*6*_, and between *Y*_*EP*_ and *F*_7_, respectively. Namely, the higher concentrations of yeast extract, MgSO_4,_ and KH_2_PO_4_ in the tested range of concentration, the higher Y_EP_. The coefficients of F_4_ and F_5_ less than 0 indicated that negative correlations existed between *Y*_*EP*_ and *F*_*4*_, and between *Y*_*EP*_ and *F*_5_, respectively. Namely, the lower concentrations of peptone and ZnSO_4_ in the tested range of concentration, the higher Y_EP_.

The coefficients of *F*_*1*_^*2*^ and *F*_*2*_^*2*^ less than 0 exhibited that there was an inverted-U-shaped relationship between Y_EP_ and F_1_, and between Y_EP_ and F_2_, and Y_EP_ had the maximum value. The following two equations were obtained by taking the derivation of Y_EP_ to F_1_ and F_2._

3 $${Y}_{EP}^{^{\prime}}=-0.118{F}_{1}+8.617$$

4 $${Y}_{EP}^{^{\prime}}=-12.468{F}_{2}+50.605$$

Suppose that *Y*_*EP*_*’* = 0, F_1_ = 8.617/0.118 = 73 h, F_2_ = 50.605/12.468 = 4.06. When the fermentation time was 73 h and glycerol concentration was 4%, the Y_EP_ yield reached the maximum value.

To sum up, the optimum fermentation medium of EP contained (g/L): 40 glycerol, 6.5 yeast extract, 2 peptone, 2.4 KH_2_PO_4_, 2.4 MgSO_4_, and 0.05 ZnSO_4_. The optimum fermentation time was 73 h. To verify the optimization efficiency, an experiment was conducted with the basal fermentation medium and optimum fermentation medium, and the fermentation time was 80 h. Under the above-mentioned conditions, the maximum theoretical yield of EP is 203.92 μg/L at 80 h. Actually, the measured EP yield with the optimum medium was 256 µg/L and significantly higher compared with the basal medium (43 µg/L) and the maximum theoretical yield.

### Kinetic model of growth

**Figure 4:**
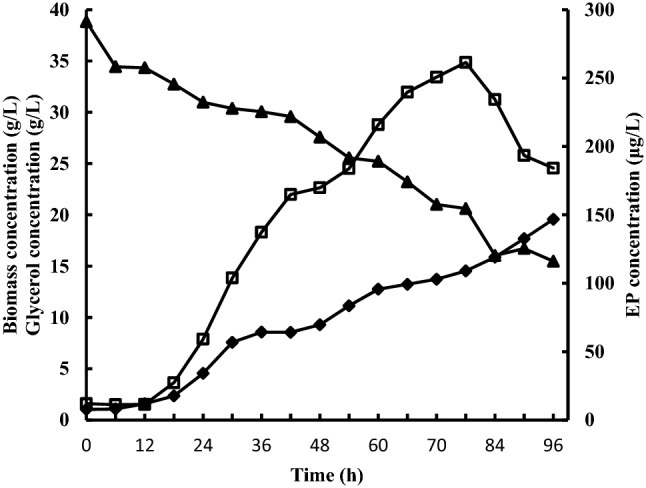
Changing profiles of biomass, EP, and glycerol consumption at different time points. ♦: Biomass concentration; ▲: Glycerol concentration; □: EP concentration.

Figure 4 shows the changing profiles of biomass, EP, and substrate in the process of *P.* *cicadae* fermentation. The biomass was increased after 12 h, and then it continued to increase rapidly till the end of the growth phase. The EP production was rapidly increased from 12 to 76 h. The maximum EP yield was obtained at 76 h (261.47 μg/L). After 76 h, the EP yield was rapidly decreased (Fig. 4). In the process of fermentation, the glycerol concentration was decreased rapidly from 34.34 to 21.02 g/L.

The experimental data of glycerol concentrations in Fig. 4 were fit with Eqs. (9)–(12), respectively (Table 3). The goodness-of-fit R^2^ values (> 0.98), F-values (> 240), and *p*-value (0.000) exhibited that these four models simulated better the kinetic experimental data, described the time course profile of EP productivity, and had the high explanation rate and credibility.

**Table 3:** The results of kinetic equation fitting of viscous fermentation broth and the results of ANOVA.

Item	Monod model			Viscosity inhibition model			Substrate inhibition model			Production inhibition model		
	Co-efficient	t-value	Sig	Co-efficient	t-value	Sig	Co-efficient	t-value	Sig	Co-efficient	t-value	Sig
Ln[C_S,0_/(C_S,0_-C_s,t_)]	37.308	3.158	0.008	–	–	–	− 34.141	5.949	0.000	–	–	–
Ln(C_S,0_-C_s,t_)	3.167	0.183	0.858	34.141	5.945	0.000	–	–	–	14.404	0.727	0.481
Ln C_s,t_	–	–	–	37.308	3.158	0.008	− 3.167	0.183	0.858	51.877	0.599	0.560
C_s,t_	–	–	–	–	–	–	–	–	–	− 10.711	1.040	0.319
R^2^	0.983			0.983			0.983			0.984		
Adj-R^2^	0.980			0.980			0.980			0.980		
F-value	371.8			371.8			371.754			249.745		
*p*-value	0.000			0.000			0.000			0.000		

Monod and logistic models are adopted as the most applicable models for cell growth. However, in addition to the limitation of nitrogen and phosphorus sources, there is an oxygen limitation in aerobic fermentation. The Monod model is generally constrained due to the presence of oxygen limitation in the aerobic fermentation process. In contrast, the logistic equation can well reflect the relationship between the cell growth rate and the biomass. In fact, in the aerobic fermentation process, the oxygen concentration is steeply decreased in the initial stage of fermentation, and then the oxygen concentration remains constant till the strain begins to die. Therefore, before the strain begins to die, the effect of oxygen concentration on cell growth can be neglected. Although the logistic model can simulate the cell growth of fungi^43,44^, the model gives very little valuable information. The combination of these four models can evaluate the reasonableness of any parameter changes in the process of fermentation.

Table 3 shows that although the *p*-value of the four models was 0.000, the F-value of the Aibe model was lower compared with the other models. Based on the F-value, we deduced that the effect of the product on the EP concentration was less compared with the viscosity and substrate. Here, it should be pointed that the product was not only EP but also other metabolites that might inhibit the synthesis of EP. To decrease the effects of viscosity and substrate on EP synthesis in the fermentation process and to increase the EP yield, fed-batch glycerol and water supplementation were used to increase the EP yield. Furthermore, water supplements could reduce the inhibitory action of production on biomass growth.

To verify our deduction, we designed three groups of experiments, and each experiment was carried out in triplicate. The EP yield of group 3 (glycerol supplementation) was 327 ± 4.93 μg/L, which was significantly higher compared with group 2 (water supplementation, 286 ± 3.23 μg/L). The EP concentration of group 2 and group 3 was markedly higher compared with group 1 (no supplementation, 272 ± 1.63 μg/L). The result further proved that the combination of four structured models could guide the optimization of the fermentation process.

## Materials and methodology

### Chemicals and microorganisms

The EP standard was purchased from Yunnan Xuli Biological Technical Co., Ltd. Methanol of the chromatographic grade was obtained from Thermo Fisher Technology Co., Ltd. Other reagents were of analytical grade or reagent grade and provided by the Sinopharm Chemical Reagent Co., Ltd., Shanghai, China.

*P.* *cicadae* strain was purchased by the China Microbiological Culture Collection Center (NO. bio-33088), maintained on the new potato dextrose agar (PDA) slant, stored at 4 °C, and passaged once every 3 months.

### Culture medium

The seed medium was a PDA medium composed of (g/L) 20 glucose, 4 yeast powder, 3 peptone, 1 KH_2_PO_4_, and 1 MgSO_4_. The basal fermentation medium was composed of (g/L) 200 fresh potato, 40 glucose, 4 peptone, 1 KH_2_PO_4_, and 2 MgSO_4_. All media were sterilized at 121 °C for 30 min in an autoclave.

### Inoculation and fermentation

Erlenmeyer flasks (250 mL) containing 100 mL seed medium were inoculated and incubated in a rotary shaker at 25 °C and 120 rpm for 72 h to prepare the inoculums. A 10% (v/v) inoculum was aseptically inoculated to 100 mL fermentation medium. The fermentation was carried out in 250-mL Erlenmeyer flasks in a rotary shaker at 25 °C and 120 rpm for 48 h. The cultured samples were centrifuged (6000×*g* for 15 min, 4 °C) to precipitate the biomass. The biomass was dried to constant weight at 80 °C for EP analysis.

### Experiment design

#### The mono-factor at a time

Growth and EP production were studied with glucose, sucrose, maltose, fructose mannitol, or glycerol as a carbon source at a concentration of 20 g/L (with the other constituents same as those in the basal fermentation medium). Different nitrogen sources [peptone, urea, yeast extract, NH_3_·H_2_O, and (NH_4_)_2_SO_4_] were implemented to basal fermentation medium at a concentration of 3 g/L, The medium without nitrogen source was used as the control. The tested inorganic salts included (g/L) KH_2_PO_4_ (1), MgSO_4_ (1), (mg /L) MnSO_4_ (20), CuSO_4_ (20), ZnSO_4_ (20), and FeSO_4_ (20). The cultures were incubated in a rotary shaker at 25 °C and 120 rpm for 48 h. After 48 h, the flasks were harvested to analyze the dry cell weight and EP yield.

#### Uniform design

Optimization studies were carried out based on a uniform design. Six independent variables were the concentrations of glycerol, yeast powder, peptone, ZnSO_4_, MgSO_4,_ and KH_2_PO_4_. Sampling time was used as the control variable. Each independent variable was assessed at 10 different levels, resulting in a U_10_(10)^6^ (Table 1). All experiments were carried out in 250-mL Erlenmeyer flasks containing 100 mL medium. The cultures were carried out in a rotary shaker at 25 °C and 120 rpm for 48 h. Three flasks of each experiment were harvested at 28, 56, 84, and 112 h to analyze dry cell weight and EP yield.

### Kinetic model

Two types of models (such as structured and unstructured models) were applied to describe a microbial process. Compared with the unstructured model, a structured model reveals more information on physiological characterization, composition, and regulatory adaptations to the environment of the microorganisms^45^. The viscous fermentation broth model (Contois model), substrate inhibition model (Andrews model), and product inhibition model (Aibe model) are a partial link between cell growth and viscosity of fermentation broth, substrate concentration, or product concentration, respectively. These models were used in our work.

#### Model assumptions

Kinetic models of microbial growth were carried out based on the Monod model, Andrews model, Contois model, and Aibe model. To describe the effect of glycerol concentration on biomass growth, the following considerations were assumed^46^.

1. The mycelium ball is thought to be a tiny chemical reactor. In the reactor, the substrate is converted into mycelia by a complex network of enzyme-catalyzed reactions. In this process, there is no mass transfer resistance inside the mycelium ball.
2. In the complex network of enzyme-catalyzed reactions, glycerol is the only limiting substrate. In all metabolic pathways converting glycerol into mycelia and metabolite, only one pathway is the limiting pathway, in which the velocity is the slowest. In all reactions of the limiting pathway, only one key step reaction controls the whole velocity of the limiting pathway. Namely, the biomass growth velocity depends on the enzyme reaction of the key step and the effect of the glycerol concentration on the limiting reaction rate.
3. The enzyme concentration of the limiting reaction is proportional to the concentration of the mycelium ball, and the concentration of the mycelium ball is proportional to the consumption concentration of the limiting substrate. The growth velocity of the mycelium ball is proportional to the consumption velocity of the limiting substrate, and the concentration of the product is proportional to the consumption concentration of the limiting substrate.

Based on these assumptions, the Monod model, Andrews model (inhibition model by substrate), Contois model (inhibition model by viscosity), and Aibe model (inhibition model by production) can be used to fit the biomass data in the progress of *P.* *cicadae.*

Monod model, Contois model, Andrews model, and Aibe model were expressed by the following equations, respectively:

5 $$\mu ={\mu }_{max}\frac{{C}_{s}}{{k}_{s}+{C}_{S}}$$

6 $$\mu ={\mu }_{max}\frac{{C}_{s}}{{k}_{s}X+{C}_{S}}$$

7 $$\mu ={\mu }_{max}\frac{{C}_{s}}{{k}_{s}+{C}_{s}+\frac{{C}_{s}^{2}}{{k}_{i}}}$$

8 $$\mu ={\mu }_{max}\frac{{C}_{s}}{{k}_{s}+{C}_{s}}+\frac{{k}_{p}}{{k}_{p}+{C}_{p}}$$

In these models, *μ* and μ_max_ are the specific growth rate and the maximum specific growth rate (h^−1^), respectively; *C*_*s*_ is the substrate concentration, namely glycerol concentration; *k*_*s*_ is the saturation constant (the substrate concentration at the half of the maximum specific growth rate, mM), *X* is the biomass concentration, *k*_*i*_ and *k*_*p*_ are the inhibition constants of substrate and inhibition product (mM), respectively, and *C*_*p*_ is the concentration of the inhibition product.

According to the definition of specific growth rate, *μ* can be expressed by the following equation

9 $$\mu =\frac{1}{X}\times \frac{dX}{dt}$$

where dX/dt is the biomass growth rate.

According to the above-mentioned assumptions, there are the following equations:

10 $$\frac{dX}{dt}=-{Y}_{b}\frac{d{C}_{S}}{dt}$$

11 $$X={Y}_{b}\times ({C}_{0}-{C}_{s})$$

12 $${C}_{p}={Y}_{p}\times ({C}_{0}-{C}_{s})$$

where *Y*_*b*_ and *Y*_*p*_ are the biomass and product yield coefficients on the substrate (glycerol), respectively.

After substituting Eqs. (5)–(8) in Eqs. (1)–(4), followed by integration and rearrangement, the following new Monod model (Eq. 9), Contois model (Eq. 10), Andrews model (Eq. 11), and Aibe model (Eq. 12) were given.

13 $$-\frac{{k}_{s}}{{C}_{0}{\mu }_{max}}\mathrm{ln}\frac{{C}_{s}}{{C}_{0}{-C}_{s}}+\frac{1}{{\mu }_{max}}\mathrm{ln}\left({C}_{0}{-C}_{s}\right)+{C}_{constant}=t$$

14 $$-\frac{{k}_{s}\times {Y}_{b}}{{\mu }_{max}}\mathrm{ln}{C}_{s}+\frac{1}{{\mu }_{max}}\mathrm{ln}\left({C}_{0}{-C}_{s}\right)+{C}_{constant}=t$$

15 $$-\frac{{k}_{s}}{{C}_{0}{\mu }_{max}}\mathrm{ln}\frac{{C}_{s}}{{C}_{0}{-C}_{s}}+\left(1+\frac{{C}_{0}}{{k}_{i}}\right)\frac{1}{{\mu }_{max}}\mathrm{ln}\left({C}_{0}{-C}_{s}\right)-\frac{1}{{k}_{i}{\mu }_{max}}{C}_{s}+{C}_{constant}=t$$

16 $$-\frac{{k}_{s}}{{C}_{0}{\mu }_{max}}\mathrm{ln}\frac{{C}_{s}}{{C}_{0}{-C}_{s}}+\frac{1}{{\mu }_{max}}\mathrm{ln}\left({C}_{0}{-C}_{s}\right)-\frac{{Y}_{p}{k}_{s}}{{\mu }_{max}{k}_{p}}\mathrm{ln}{C}_{s}-\frac{{Y}_{p}}{{\mu }_{max}{k}_{p}}{C}_{s}+{C}_{constant}=t$$

These equations were applied to fit the data of kinetic experiments.

#### The model solution and simulations

Experimental data from batch fermentation in a 20-L mechanically stirred fermentor were utilized to simulate the kinetic parameters by the developed model Eqs. (9)–(12). The fermentation medium was the optimized medium. The fermentation conditions of the 20-L mechanically stirred fermentor were set as follows: medium volume 15 L, inoculum 10%, rotational speed 120 rpm, aeration rate 15 L/min, and temperature 25 °C.

#### Verification of kinetic inference

The verification tests were carried in a 20-L mechanically stirred fermentor. The three experiments were designed in agreement with the kinetic experiments. Water and glycerol were not supplied in the group 1 experiment, and 1 L water was supplied at 40 h and 60 h in the process of fermentation in the group 2 experiment. In the group 3 experiment, 900 g glycerol was added in three batches, 300 g was added to the medium before sterilization, and another 600 g glycerol (2,000 mL 30% glycerol aqueous solution) was added at 40 h and 60 h, respectively. The fermentation conditions were the same as the kinetic conditions and fermentation time 80 h.

### Analytical methods

#### Preparation of sample solution

Briefly, the biomass of *P.* *cicadae* was dried to constant weight and powdered in a grinding mill. Next, 0.10 g biomass powder was loaded into a 5-mL centrifuge tube and mixed with 3 mL MeOH. The tube was sonically extracted at 25 °C for 30 min. The conditions of ultrasonic treatment were set as follows: frequency 40 Hz and power 50 W. The tube was centrifuged at 1.3 × 10^4^ rpm for 2 min. The supernatant was subjected to LC–MS/MS analysis. The extraction was performed in triplicate.

#### *Preparation of standard EP stock solution (50* *μg/mL)*

Briefly, 10 mg standard EP was dissolved in 200 mL methanol to prepare a standard EP stock solution. Before use, the stock solution was diluted with methanol to prepare the working solutions of various concentrations.

#### Chromatographic conditions of LC–MS/MS

An HPLC–MS/MS (Agilent 1290 UPLC/6540 Q-Tof, USA) system equipped with an Acquity UPLC BEH C18 column (130 Å, 1.7 μm, 2.1 × 100 mm) was used in the present determination. A gradient elution procedure was applied to the elution of EP. The mobile solvent A and solvent B used in the gradient elution procedure were 0.1% formic acid–methanol and 0.1% formic acid–water, respectively. The elution procedure was programmed as follows: 10% A to 95% A (0–2.5 min), 95% A to 95% A (2.5–10 min), 95% A to 10% A (10–10.5 min), and 10% A to 10% A (10.5–12.5 min). Other conditions were set as follows: flow rate 0.2 mL/min, column temperature 40 °C, injection volume 5 μL, and sample temperature 20 °C. The quantization was operated in the multi-response monitoring (MRM) mode with a positive ion mode to monitor the precursor-production ion pair transitions of m/z 429.6–393.6 for EP.

### Data analysis

Non-linear multiple regression analyses have been utilized in the optimization of medium components and the parameters of reaction process to find the optimum medium and to determine the optimum reaction process, which often seem to provide higher accuracy^47,48^^.^ The p-value, F-value and R^2^ derived from Statistical Computing software were used to apply multi-variable non-linearregression analysis. In the study, after a series of modeling, according tot p-value, F-value and R^2^, the optimum medium and reaction process were found.

One way analysis of variance (Anova) is a method to analyze the results of univariate analysis and to test whether test factors have a significant effect on test results. One-way ANOVA has proven its effectiveness in solving the problem of high dimensionality in the feature space^49^. Thus, the one way Anova was applied into the study as a filter method to select the relevant features.

All data were expressed as means ± standard errors (in the mono-factor at time and the Uniform-design experimentation) or as means (in the kinetic model experiment). All the multi-ple non-linear regression equations were conducted using SPSS 17.0 software (IBM, Ammonst City, USA) and Microsoft Excel (Microsoft, Redmond City, USA), the analysis of variance (ANOVA) was performed by Dunnett's test, and p < 0.05 was considered as statistically significant.

## Conclusions

The optimal medium for maximum production of EP by *P.* *cicadae* was determined by a combination of a mono-factor experiment, a uniform design method, and a non-linear regression. This was the first report on EP production by *P.* *cicadae* fermentation. The optimum fermentation time was 80 h. The maximum flask culture yield of EP yield (256 μg/L) after optimization was increased by nearly five-fold compared with that before the optimization, which was also higher than the maximum theoretical EP yield (203.92 μg/L). The combinational use of four structured models indicated that glycerol and water could further increase the yield of EP in the fermentation process of *P.* *cicadae*.
